# Changing Pattern of HDV Infection in Italy Over 40 Years; the Current Scenario and the Impending Challenge

**DOI:** 10.1111/jvh.70172

**Published:** 2026-03-17

**Authors:** Gian Paolo Caviglia, Tommaso Stroffolini

**Affiliations:** ^1^ Department of Medical Sciences University of Torino Turin Italy; ^2^ Department of Tropical and Infectious Diseases Policlinico Umberto I Rome Italy

**Keywords:** chronic hepatitis D, cirrhosis, epidemiology

## Abstract

Since the initial description of HDV, in the last four decades Italy has witnessed a profound decline of the infection driven by HBV vaccination, contrasted however by the reconstitution of a viral reservoir through migratory flows from endemic HDV areas; in parallel, the medical scenario has changed, resulting in different clinical outcomes. The epidemiological and clinical changes were documented by national surveys performed up to 2025, which provided an ongoing perspective on the long‐term evolution of HDV, highlighting the temporal trends in prevalence, risk factors, and clinical features. This review summarises the changes over time of HDV in Italy and the contemporary epidemiologic features of an infection that is vanishing among natives but increasing among migrants, outlining the medical challenge of a new heterogeneous clinical spectrum, including indolent phenotypes.

**Abbreviations**
CHD: chronic hepatitis D
HBsAg: hepatitis B surface antigen
HBV: hepatitis B virus
HCC: hepatocellular carcinoma
HCV: hepatitis C virus
HDAg: hepatitis D antigen
HDV: hepatitis D virus
HIV: human immunodeficiency virus
IVDU: intravenous drug use

## 1 Recognition and Inception of the Infection, Characterisation of Hepatitis D: 1980–1995

Hepatitis Delta virus (HDV), first described in Italy in 1977 [1], is a defective, single‐stranded RNA virus, requiring hepatitis B virus surface antigen (HBsAg) envelope for assembly and transmission [2]. Its discovery significantly expanded the understanding of chronic viral liver diseases and the natural history of hepatitis B virus (HBV) infection.

When serological assays for HDV diagnosis became available in the early 1980s, the epidemiological and medical paradigms of the infection and disease were elaborated from extensive studies conducted in Italy. A 1982 study of acute HBsAg‐positive hepatitis reported a 39% prevalence of anti‐HDV among 111 patients with fulminant HBV hepatitis, a markedly higher rate than the 19% observed in 532 patients with benign acute hepatitis B [3].

A 1983 study of chronic HDV infection showed a striking 24.6% prevalence of anti‐HDV among 822 carriers with liver disease, compared with only 7.1% in 492 asymptomatic carriers [4]. Infection rates were higher in Southern than northern Italy; the mean age of HDV‐infected individuals was 34 years, with males predominating over females by approximately 5 to 2. Transmission occurred through overt parenteral exposure, particularly among people who inject drugs, many of them coinfected with hepatitis C virus (HCV) and/or human immunodeficiency virus (HIV) [5], or through covert transmission in the household [6].

A 1983 clinical/histologic study of 101 patients exhibiting the hepatitis D antigen (HDAg) in the liver showed that the predominant liver disease at presentation was chronic active hepatitis (in 93) or cirrhosis (in 32). Among 75 patients without nodular regeneration at baseline biopsy, cirrhosis developed within a mean interval of 6 years; patients with HDV‐related cirrhosis were 10 years younger than those with HBV‐related cirrhosis, and disease severity was greatest among individuals with injection drug use [7].

In 1992, a further study [8] showed a similar prevalence of anti‐HDV (23.4%) and provided a detailed analysis of risk factors associated with Delta infection. Independent predictors of anti‐HDV positivity included age 30–49 years (adj. O.R. 2.5, 95% CI 1.4–4.5), residence in southern regions (adj. O.R. 1.6; 95% CI 1.1–2.5), intravenous drug use (IVDU) (adj. O.R. 8.4, 95% CI 4.4–15.9), and cohabitation with an anti‐HDV positive subject (adj. O.R. 12.9, 95% CI 4.0–33.5). IN contrast, blood transfusion and male homosexuality were not associated with increased risk.

These seminal contributions established hepatitis D as a severe and often lethal form of viral hepatitis, marked by fulminant manifestation in acute infection and accelerated progression to cirrhosis and hepatocellular carcinoma (HCC) in chronic infection. The perception of greater clinical severity compared to hepatitis B has been consistently confirmed by subsequent studies and persists at present, influencing the medical approach to the disease.

## 2 Transition Period: 1996–2010

Subsequent surveys conducted in 1997 [9], 2000 [10], and 2008 [11] documented a significant decline in anti‐HDV prevalence (14.4%, 8.3%, and 9.7%, respectively), largely attributable to improved socioeconomic conditions, increased public awareness, and the introduction in 1991 of compulsory hepatitis B vaccination [12]. By the early 2000s, Italy had become a model of HBV/HDV control, with markedly reduced reservoirs of infection among younger generations.

Clinically, HDV appeared to maintain its severity, with cirrhosis and hepatocellular carcinoma continuing to represent common outcomes, although increasingly confined to older Italians with long‐standing infections. Genotype I of HDV remained predominant in the country [13, 14]. Nevertheless, an insidious disease course spanning several decades was observed in a subset of patients, challenging the perception of chronic hepatitis D (CHD) as an almost invariably aggressive disease [15].

## 3 A Vanishing Infection in Italians, the Increasing Challenge From Migrants: 2011–2025

The 2017 [16] and 2020 [17] surveys confirmed a stable prevalence of anti‐HDV in the general population (Table 1), alongside a continued decline among Italian‐born individuals, reinforcing the concept of HDV as a “vanishing disease”. The endemic reservoir was aging, and new infections in younger Italians had been virtually eliminated through HBV vaccine‐induced immunity.

**TABLE 1:** Prevalence (%) of anti‐HDV‐positive patients among HBsAg‐positive carriers in seven nationwide studies performed in Italy.

Year of study	N. anti‐HD+/HBsAg+	Anti‐HD prevalence (%)	References
1983	202/822	24.6%	[4]
1992	364/1556	23.4%	[8]
1997	149/990	14.4%	[9]
2000	69/834	8.3%	[10]
2008	86/887	9.7%	[11]
2017	61/513	11.9%	[16]
2020	78/786	9.9%	[17]

Abbreviations: HBsAg, hepatitis B surface antigen; HD, hepatitis D.

The national HBV vaccination campaign introduced in 1991 and targeting 3‐month‐old infants as well as 12‐year‐old adolescents for the first 12 years of implementation has generated a nearly complete cohort of Italian population under 45 years of age who are immune to HBV [18]. The vaccine also controlled HDV by decreasing the pool of HBV chronic carriers, who represent the biological substrate for HDV spreading.

Impressive clinical and epidemiological changes have emerged over time when comparing the first survey with full data available in 1992 [8] to the most recent in 2025 [19]. The sex ratio decreased by half (from 3.1 to 1.6; *p* < 0.001), while mean age increased from 38.7 to 55.1 years (*p* < 0.001). The proportion of subjects reporting intravenous drug use (IVDU), which represents the worldwide leading mode of HDV spreading [20], declined markedly, as did the proportion reporting cohabitation with an anti‐HDV‐positive individual, a well‐established risk factor for intrafamilial spread [6]. Both measures were reduced by more than 50% (from 26.9% to 12.7% and from 19.1% to 9.1%, respectively). In contrast, the proportion of subjects with liver cirrhosis among all HDV‐positive cases rose sharply from 35.7% to 62.6% (*p* < 0.001) (Table 2).

**TABLE 2:** Changing pattern of some characteristics of people with chronic HDV infection (1992–2024).

Characteristic	1992 [8]	2025 [19]	*p*
Sex ratio (M/F)	3.1	1.6	< 0.001
Mean age (years)	38.1	55.1	< 0.001
Reporting IVDU	26.9%	12.6%	< 0.001
Household contact of an HDV+ subject	19.1%	9.1%	< 0.001
Presence of cirrhosis	35.7%	62.6%	< 0.001

Abbreviations: F, female; HDV, hepatitis Delta virus; IVDU, intravenous drug use; M, male.

**FIGURE 1:**
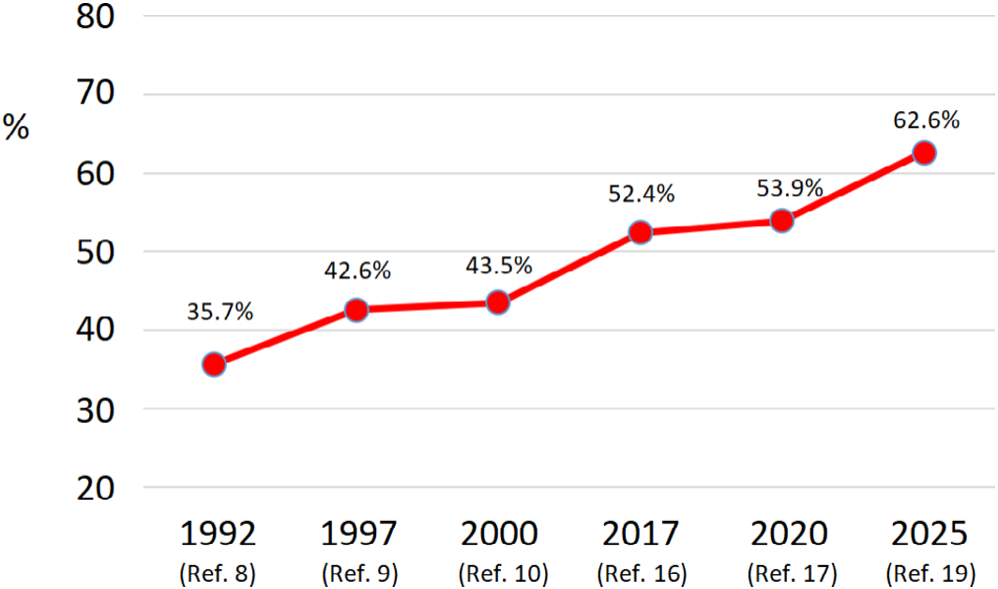
Temporal trend (1992–2025) of the proportion (%) of subjects with HDV‐related liver cirrhosis among those with chronic HDV infection in Italy. In the 1983 and 2008 studies, the information was lacking.

The progressive increase of liver cirrhosis followed a linear trend across the study period (Figure 1), likely reflecting a “survival effect”, whereby an accumulating cohort of old subjects with long‐standing chronic HDV infection, acquired decades earlier, now present with advanced disease. Indeed, HDV infection has been shown to persist for several decades with a relatively indolent course in a substantial subset of cases [15].

The remarkable decline by more than 50% from 1992 to 2024 in reported exposure to the two main HDV transmission routes (IVDU and household contact with an HDV‐positive subject) demonstrates effective control of domestic transmission dynamics. These favourable findings reflect the immune status against HBV, and consequently against HDV, achieved in younger Italian cohorts through widespread vaccination. Among native Italians, HDV can therefore be considered a largely vanishing infection.

Migratory flows operate in an opposite direction. As of 1 January 2025, the number of regular migrants (i.e., those with legal residence permits granting full access to healthcare) living in Italy reached 5,307,538, corresponding to 9.0% of the Italian resident population [21]. Many migrants originate from countries with high HDV prevalence, where infection is estimated to affect 3%–6% of chronic HBsAg carriers [22] (Table 3). These figures likely underestimate the true burden due to limited diagnostic availability and structural barriers to healthcare access. A recent survey has identified localised clusters with very high HDV prevalence across Sub‐Saharan Africa, both in Western and Central regions [23].

**TABLE 3:** Proportion of migrants legally resident in Italy in 2024 and of those anti‐HDV‐positive in the 2024 survey by area of provenience (Reference [10]). Estimated anti‐HD prevalence in their area of provenience.

Area of provenience	Proportion of migrants legally residing in Italy(total number 5,307,538) [21]	Proportion of migrants anti‐HDV+ residing in Italy in 2025 survey (total number 198) [19]	Estimated anti‐HDV prevalence in their area of provenience [22]
Eastern Europe and former U.R.S.S.	46.2%	85.9%^a^	3.0% (2.1–4.2)
Africa	22.7%	9.6%	6.0% (5.0–7.2)
Asia	23.4%	3.0%	3.2% (0.4–12.4)
Central‐South America	7.6%	1.0%	5.9% (3.0–9.7)

Abbreviation: HDV, hepatitis Delta virus.

^a^ Of them, 41.8% from Romania, 34.7% from Moldova, 14.7% from Albania, 6.5% from Ukraine, 1.2% from Georgia, and 1.2% from Russia.

In Italy, the proportion of anti‐HDV‐positive migrants has increased nearly threefold over the past decade, from 13.3% in 2017 [16] to 38.4% in 2025 [19]. Similar patterns have been documented across Western Europe, including Germany [24], Greece [25], France [26], and the United Kingdom [27], where migrants from Turkey, Eastern Europe, the former Soviet Union, Africa, and the Middle East substantially contribute to the current pool of HDV‐infected subjects.

## 4 Acute HDV Infection

Incidence data on acute Delta hepatitis are generally scarce worldwide. In Italy, however, a surveillance system for monitoring the incidence and risk factors for all types of acute viral hepatitis has been active since 1987. According to these data, the incidence rate of acute Delta hepatitis declined from 3.2 cases per 1 million population in 1987 to 0.04 in 2019, making it a rare occurrence today [28]. This downward trend parallels the decline in acute hepatitis B, which fell from 18.0 to 0.39 cases per 100,000 population over the same period [17], reflecting the effectiveness of the national HBV vaccination campaign in controlling both viruses.

A comparison of acute HDV cases two decades apart (1991–1991 vs. 2011–2019) reveals significant epidemiological shifts: a growing proportion of infected females (sex ratio 3.8 vs. 2.1), an older median age (21 years vs. 44 years), and an increasing proportion of coinfections (55% vs. 75%). The proportion of individuals reporting IVDU, still the leading HDV transmission route globally [20], showed a ten‐fold decrease (45.0% vs. 4.2%). Among acute HDV cases, the proportion of migrants (data available since 2004) increased from 24.6% in 2004–2010 to 32.1% in 2011–2019. Notably, none of the migrant cases reported IVDU, whereas 33.3% reported high‐risk sexual exposure.

These findings mirror the epidemiological patterns observed among individuals with chronic HDV infection, indicating that acute HDV has become a rare disease in Italy. However, its increasing incidence among migrants, particularly with one third of cases linked to risky sexual behaviour, highlights the need for careful monitoring in the years to come.

## 5 Coinfection HDV/HIV

Coinfection with HDV is common in individuals infected with immunodeficiency virus (HIV) due to similarities in modes of transmission (especially intravenous drug use). In Italy, the prevalence of anti‐HDV among HIV positive subjects is 18.8%, mostly intravenous drug users; however, the prevalence declined from 28% in the cohort enrolled during the years 1997–1998 to 12% in that enrolled during the years 2012–2015 [29]. The triple threat (HBV/HDV/HIV coinfection) has become the leading cause of death and liver decompensation in HIV‐infected patients receiving antiretroviral therapy [30].

## 6 Is the Clinical Paradigm Changing?

An extended and comprehensive nationwide survey of HBsAg carriers with anti‐HDV positivity has been recently published [19]. The study enrolled 515 patients from 32 Italian centres during the years 2022–2025, and primarily compared the demographic and clinical features of native Italians and migrants.

Italians predominated (61.6% versus 38.4%), largely because participating centres followed a higher number of long‐standing Italian patients compared with newly diagnosed migrants. Independent predictors of liver cirrhosis included age > 50 years (O.R. 1.72; 95% CI 1.09–2.74), birth in Italy (O.R. 1.77; 95% CI 1.13–2.80), and HDV‐RNA levels > 3 Log IU/mL (O.R. 1.92; 95% CI 1.31–2.81). Overall, liver cirrhosis was present in nearly two‐thirds (62.7%) of cases. HDV‐RNA was detectable in 83.9% of subjects, and genotype 1 accounted for > 99% of HDV infections.

Italian patients were older (median age ~60 years), predominantly male, and more frequently diagnosed with cirrhosis (70.3%) and HCC (14.8%). Migrants were younger (median age ~46 years) and more often female. Notably, both groups exhibited a broader clinical spectrum than traditionally perceived, ranging from active CHD to milder inactive/minimal disease phenotypes.

Importantly, cirrhosis was also common among migrants (50.5%), suggesting either delayed diagnosis or rapid disease progression in selected individuals. The latter finding appears to modify our understanding of CHD as a severe and progressive disease. Among Italians exposed to the HDV decades before, non‐cirrhotic CHD often followed a slow, indolent course, indicating that insidious forms of the disease are more frequent than previously recognized.

## 7 Summary of Changing Clinical and Virological Features

Several notable trends have emerged over the past four decades. Among Italians, the mean age of HDV‐infected patients has increased, while the historical male predominance has diminished. Both IVDU and intrafamilial transmission have declined twofold, reflecting the impact of HBV vaccination and the reduction of domestic reservoirs of infection. The proportion of patients with cirrhosis has nearly doubled, likely representing a survival effect in older cohorts with long‐standing infection. Meanwhile, migrants now constitute a substantial share of HDV cases, fundamentally reshaping the epidemiological landscape.

## 8 The Future Scenario

Italy represents a paradigm of HDV evolution in a high‐income country where two key factors operate in opposite directions: extensive HBV vaccination coverage and increasing migratory flows. Among native Italians, HDV can now be regarded as a vanishing infection: younger generations are largely immune, and the remaining infected population is progressively aging. Conversely, among migrants, HDV is a refreshing disease, reconstituting the viral reservoir and sustaining healthcare needs.

Although migrants are progressively replacing native Italians in the pool of subjects with HDV infection, transmission to Italians is unlikely, as the latter are largely immune to HBV and therefore no longer at risk of acquiring HDV. Notably, however, one‐third of migrants with acute HDV acquire the infection in Italy through high‐risk sexual exposure.

The opposing effects of these two key forces are expected to lead to the near disappearance of HDV among native Italians. This turning point will be driven by the expanding proportion of HBV‐vaccinated Italian cohorts and the gradual loss, due to mortality, of older Italian patients with long‐standing HDV‐related cirrhosis. Conversely, HDV infection in migrants may represent a new challenge in the years to come.

Continued progress towards HDV control will require tailored strategies, including:

- Continued surveillance and reflex testing for anti‐HDV in all HBsAg‐positive individuals, with particular attention to migrants [31].
- Integration of HDV screening into migrant health programs.
- Expanded access to novel therapies, such as bulevirtide, which may change the natural history of disease [32].
- Ongoing surveillance of the dynamic balance between disappearing domestic infections and persistent imported cases

## Author Contributions

Gian Paolo Caviglia and Tommaso Stroffolini: concept and design, interpretation of the data, drafting the article, and critical revision. All authors approved the final version of the manuscript.

## Funding

The authors have nothing to report.

## Ethics Statement

The authors have nothing to report.

## Conflicts of Interest

The authors declare no conflicts of interest.

## Data Availability

Data sharing not applicable to this article as no datasets were generated or analysed during the current study.
